# Effects of rifaximin on the fecal microbiota of healthy dogs: a pilot study

**DOI:** 10.3389/fvets.2026.1761247

**Published:** 2026-10-23

**Authors:** Hiroki Harada, Masahiro Tamura, Maki Kawabata, Atsushi Sakatani, Tatsuya Deguchi, Akira Fukuda, Masaru Usui, Hiroshi Ohta

**Affiliations:** 1 Companion Animal Internal Medicine, Department of Companion Animal Clinical Sciences, School of Veterinary Medicine, Rakuno Gakuen University, Ebetsu, Hokkaido, Japan; 2 Food Microbiology and Food Safety Unit, Division of Preventive Veterinary Medicine, School of Veterinary Medicine, Rakuno Gakuen University, Ebetsu, Hokkaido, Japan

**Keywords:** 16S rRNA, antibiotics, dysbiosis, gut microbiota, healthy dogs, rifaximin

## Abstract

**Introduction:**

Rifaximin is a poorly absorbed antibiotic that is used for the treatment of hepatic encephalopathy in humans. However, it is important to evaluate the effects of antibiotics on the gut microbiota because antibiotics may alter gut microbial composition. This study aimed to investigate the effects of 28 days rifaximin administration on the gut microbiota of healthy dogs.

**Methods:**

Five healthy dogs were administered rifaximin (25 mg/kg q12h) for 28 days. Fecal samples were collected from the rectum on day 0 (before rifaximin administration), day 14, and day 28. Fecal microbiota composition and diversity were evaluated by 16S rRNA gene sequencing targeting the V3–V4 region at three time points.

**Results:**

No adverse effects, including gastrointestinal symptoms, were observed during the administration. Firmicutes was the most abundant phylum at all time points, while Fusobacteriota, Bacteroidota, and Actinobacteriota were also major phyla. No significant changes were observed in alpha diversity metrics, including amplicon sequence variants (*p* = 0.449), Chao1 (*p* = 0.241), and the Shannon index (*p* = 0.248), and beta diversity metrics, including weighted UniFrac distances (*p* = 0.471), unweighted UniFrac distances (*p* = 0.157), and Bray–Curtis dissimilarities (*p* = 0.089).

**Conclusion:**

No statistically significant changes in fecal microbiota alpha or beta diversity were detected following 28 days of rifaximin administration. Given the small sample size and lack of an untreated control group, these findings should be interpreted as preliminary and do not provide evidence to guide clinical use of rifaximin in dogs.

## 1 Introduction

The gut–liver axis is a bidirectional relationship between the gastrointestinal tract and the liver, resulting from complex interactions among multiple factors (1–3). The intestinal mucosal barrier functions as an anatomical filtering system that removes gut-derived toxins and microbial components, and the residual toxins are transported to the liver via the portal vein for detoxification. In return, the liver secretes bile acids and antibodies into the duodenum, which helps maintain mucosal homeostasis by suppressing small intestinal bacterial overgrowth (SIBO) and inhibiting bacterial adhesion to the epithelium (4, 5).

Hepatic encephalopathy (HE) is a neurological disorder caused mainly by ammonia produced by gut bacteria. However, in humans, recent advances in bioinformatics and rapid progress in microbiome research have revealed that patients with HE have dysbiosis, which is defined as a reduction in beneficial gut bacteria and an overgrowth of harmful bacteria (6, 7). Furthermore, dysbiosis exacerbates HE by elevating endotoxin levels and damaging the intestinal mucosal barrier (8). Therefore, appropriate modulation of the gut microbiota is considered an important strategy for the treatment of HE in humans.

Rifaximin is a semisynthetic antibiotic derived from rifamycin that exhibits broad-spectrum activity against both Gram-positive and Gram-negative bacteria in the gut (9, 10). The absorption rate of rifaximin in the gastrointestinal tract is extremely low, with systemic absorption reported to be less than 0.4% (11). Because of these characteristics, rifaximin has traditionally been used as a gut-targeted antibiotic for various gastrointestinal disorders in humans (12–14). More recently, rifaximin was approved by the U.S. Food and Drug Administration to reduce the risk of recurrence in patients with overt HE (15). Rifaximin reduces ammonia and endotoxin levels by suppressing ammonia-producing bacteria and improving cognitive function in patients with HE. Unlike other antibiotics, numerous studies have shown that its effects are largely limited to specific pathogens, with minimal impact on healthy gut microbiota (16–18).

In veterinary medicine, HE is managed using a multimodal approach that combines a protein-restricted diet, lactulose, and antibiotics such as metronidazole (19, 20). However, the efficacy of antibiotics in the treatment of HE remains unclear, and their use is discouraged because of potential adverse effects and antimicrobial resistance (21). Serrano et al. investigated the potential benefits of metronidazole in dogs with congenital extrahepatic portosystemic shunts (EHPSS), which are a major cause of HE (22). No additional benefit of metronidazole was observed, suggesting that hepatic-supportive diets combined with lactulose are sufficient for the presurgical medical management of dogs with EHPSS. Favier et al. reported an estimated median survival time of 3.2 years in dogs with congenital portosystemic shunts (CPSS) that did not undergo surgery and were managed medically with a protein-restricted diet or lactulose (23). In this cohort, survival times ranged from long-term survivors to dogs that died within a relatively short period. Therefore, although dietary management and lactulose administration are effective in controlling HE in many dogs, they may be insufficient in some cases, and additional therapeutic options are needed. In humans with HE, a combination of rifaximin and lactulose has been shown to achieve high clinical efficacy, which may benefit the management of HE in dogs.

However, many antibiotics may adversely affect the diversity and composition of the gut microbiota and disrupt the homeostasis of the gut-liver axis (24–26). Metronidazole induces reversible alterations in the gut microbiota of healthy dogs (25). In this context, rifaximin has the potential to provide therapeutic benefits for HE while preserving the gut microbiota. Therefore, this pilot study aimed to describe fecal microbiota profiles before and during 28 days of rifaximin administration in healthy dogs, as preliminary information for future controlled studies in canine clinical populations.

## 2 Materials and methods

### 2.1 Dogs and experimental design

Five healthy laboratory beagles (two males and three females, median age 9 years [range; 8–11 years], body weight 11.2 kg [range; 10.3–14.4 kg]) were enrolled in this study. All dogs were maintained at a single facility affiliated with our university. The dogs were fed the same commercially available dry food from 1 month before the experiment through the 28-day study period and had not received any antibiotics, anthelmintics, or antidiarrheal agents within the preceding 6 months. All dogs were considered clinically healthy based on the absence of gastrointestinal signs, physical examination results, complete blood counts (CBC), and serum biochemistry (albumin, total bilirubin, aspartate aminotransferase, alanine aminotransferase, alkaline phosphatase, lipase, blood urea nitrogen, creatinine, glucose, total bile acids, ammonia). The dose of rifaximin was set at 25 mg/kg q12h for 28 days and was administered orally based on a previous study demonstrating the clinical efficacy of rifaximin in dogs with chronic enteropathy (27). All administrations were performed by the same investigator, and rifaximin was administered orally with a small amount of water after feeding. Successful administration was confirmed by visual inspection of the oral cavity immediately after dosing. Dogs were subsequently monitored for at least 15 min to confirm the absence of vomiting. Fecal samples were collected by digital rectal examination on day 0 (before administration; D0), day 14 (D14), and day 28 (D28), immediately placed into sealed tubes, and stored at −20 °C until analysis. All animal procedures were approved by the Animal Care and Use Committee of our university (No. VH23B8, approved on June 23, 2023).

### 2.2 16S rRNA sequencing and data analysis

Approximately 0.2 g of feces was homogenized using Lysing Matrix B (MP Biomedicals, LLC, Irvine, CA, USA) and a MagNA Lyser (Roche Diagnostics K.K., Tokyo, Japan). Fecal DNA was extracted following the manufacturer’s protocol for ISOFECAL for Beads Beating (NIPPON GENE Co., Ltd., Tokyo, Japan). The V3–V4 region of the bacterial 16S rRNA gene was amplified by polymerase chain reaction using the 341F (5′-TCGTCGGCAGCGTCAGATGTGTATAAGAGACAGCCTACGGGNGGCWGCAG-3′) and 806R (5′GTCTCGTGGGCTCGGAGATGTGTATAAGAGACAGGACTACHVGGGTATCTAATCC-3′) primers following the 16S Metagenomics Sequencing Library Preparation at 95 °C for 3 min, followed by 25 cycles at 94 °C for 45 s, 50 °C for 60 s, 72 °C for 90 s, and 72 °C for 10 min. The first polymerase chain reaction (PCR) mixture consisted of 0.25 μL of Takara Ex Taq HS (Takara Bio Inc., Shiga, Japan), 3.0 μL of 10 × Ex Taq Buffer (Mg^2+^ plus), 2.4 μL of dNTP mixture, 3.0 μL of each primer, 3.0 μL of template DNA, and was brought to 30 μL with DNase/RNase-free water. Negative controls without template DNA were included in the first PCR to monitor potential contamination. PCR products were purified using AMPure XP (Beckman Coulter Inc., Brea, CA, USA). The purified DNA was transported to an external laboratory (Genome-Lead Co., Ltd., Kagawa, Japan), where the second PCR was performed using KAPA HiFi HotStart ReadyMix (Kapa Biosystems, Wilmington, MA, USA) at 95 °C for 3 min, followed by 8 cycles at 95 °C for 30 s, 55 °C for 30 s, 72 °C for 30 s, and 72 °C for 5 min. After purifying the amplicons using the same procedure as in the first PCR, comprehensive sequencing was performed using an Illumina MiSeq system (Illumina, San Diego, CA, USA) with 2 × 301 bp paired-end reads. QIIME 2 software (version 2025.7) was used to analyze the 16S rRNA sequencing data. The DADA2 plugin (q2-dada2, version 2025.7) was used to denoise the sequencing data. Based on the interactive quality plots, the first 15 bases of both forward and reverse reads were trimmed and the reads were truncated at position 240 where the quality scores decreased below 30. Taxonomy was assigned using the classify-sklearn method in the q2-feature-classifier plugin with SILVA version 138 (28).

To evaluate the richness and evenness of the microbiome in each sample, alpha diversity metrics, including the Shannon index, Chao1, and amplicon sequence variants (ASVs) were calculated. To assess differences in community composition between samples, beta diversity metrics, including weighted and unweighted UniFrac distances and Bray–Curtis dissimilarities, were calculated. Beta diversity metrics were visually assessed using principal coordinate analysis (PCoA) plots, visualized in R version 4.5.3 (R Foundation for Statistical Computing, Vienna, Austria).

### 2.3 Statistical analysis

Statistical analyses were performed using QIIME 2 plugins, R, or commercial statistical software (JMP^®^ Student Edition 18.2.1). Friedman’s test was used to compare changes in alpha diversity metrics across the three time points. Permutational multivariate analysis of variance (PERMANOVA) was performed using adonis2 in the vegan R package (version 2.7.5) for the longitudinal assessment of beta diversity. Permutational analysis of multivariate dispersions (PERMDISP) was performed using betadisper to assess the homogeneity of multivariate dispersions, followed by a permutation test using permutest. Permutations were restricted within dog for both analyses to account for repeated measurements. Statistical significance was set at *p* < 0.05. Alpha and beta diversity metrics were evaluated as separate ecological measures, and no additional correction for multiple comparisons among diversity metrics was applied because such correction would not alter the conclusions when all *p*-values are >0.05.

## 3 Results

All dogs received rifaximin in accordance with the study protocol. No gastrointestinal symptoms were observed in any of the dogs during the study period. Additionally, no abnormalities were detected in serum biochemical analyses conducted every 14 days. All stool samples were well-formed, allowing accurate measurement of the amount required for DNA extraction. No amplification was observed in the PCR negative controls. All raw data were available for the microbiome analysis.

### 3.1 The composition of fecal microbiota

The results of the taxonomic analyses are shown in Figure 1. At the phylum level, the fecal microbiota mainly consisted of four phyla. In groups D0, D14, and D28, Firmicutes was the dominant phylum, while Fusobacteriota, Bacteroidota, and Actinobacteriota were also major phyla. This composition was maintained throughout the study period, and no apparent changes were observed.

**Figure 1:**
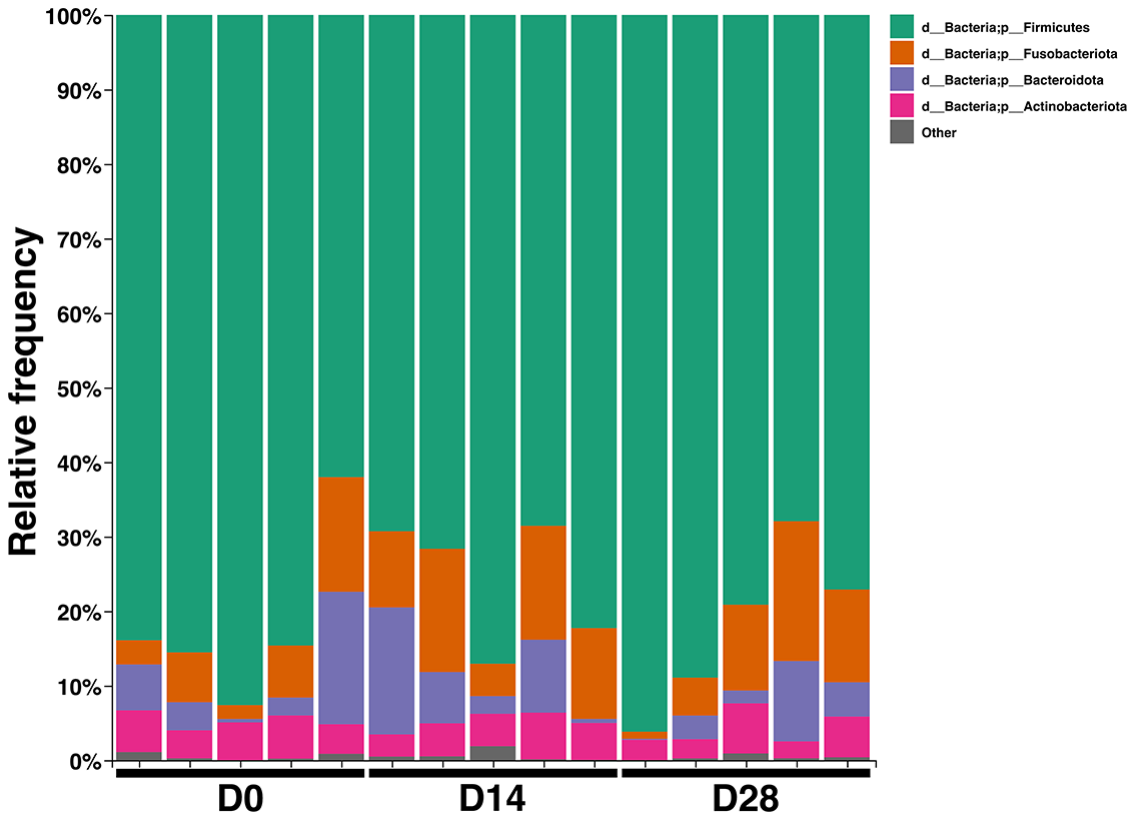
The relative abundance of fecal microbiota at the phylum level on day 0 (D0), day 14 (D14), and day 28 (D28). Firmicutes was the most dominant phylum, while Fusobacteriota, Bacteroidota, and Actinobacteriota were also major phyla. This composition was maintained during the study period.

### 3.2 The diversity of fecal microbiota

After quality filtering and denoising, the number of reads per sample ranged from 28,093 to 54,880, with a median of 37,828 reads. The per-sample DADA2 denoising statistics are provided in Supplementary file 1. To evaluate sequencing depth, we generated alpha rarefaction curves and performed sample normalization using a sampling depth determined based on the results (Supplementary file 2). The results of the alpha diversity metrics, including ASVs, Chao1, and the Shannon index, are shown in Figure 2. ASVs (*p* = 0.449) and Chao1 (*p* = 0.241) showed a decreasing trend on D14 and D28 compared with D0. In contrast, the Shannon index (*p* = 0.248) showed a decreasing trend at D14 compared with D0 but remained at a similar level at D28 compared with D14. However, no significant changes were observed in any of the alpha diversity metrics among D0, D14, and D28.

**Figure 2:**
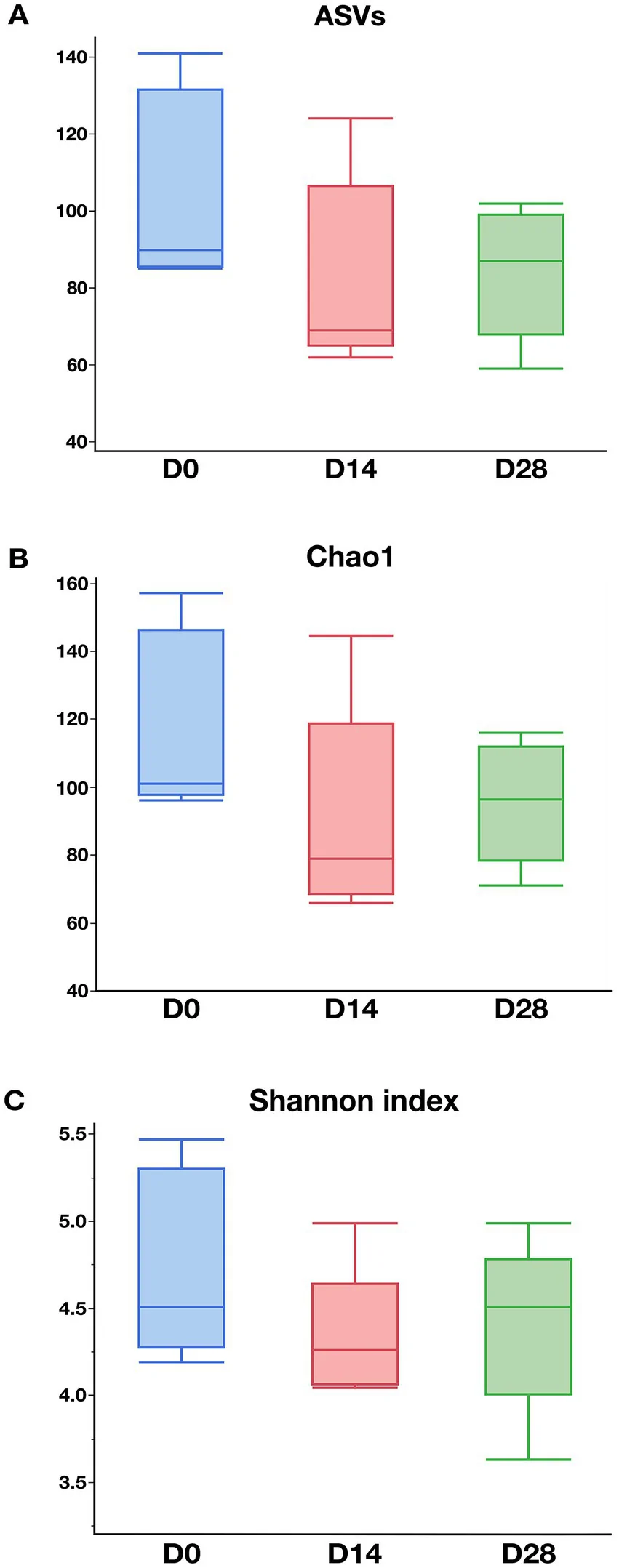
Alpha diversity of the fecal microbiota on day 0 (D0), day 14 (D14), and day 28 (D28) based on **(A)** ASVs, **(B)** Chao1, and **(C)** the Shannon index. The Friedman test was used to compare alpha diversity across the three time points. ASVs (*p* = 0.449) and Chao1 (*p* = 0.241) showed a decreasing trend at D14 and D28 compared with D0. In contrast, the Shannon index (*p* = 0.248) showed a decreasing trend at D14 compared with D0 but remained at a similar level at D28.

PCoA plots based on beta diversity metrics, including weighted UniFrac distances, unweighted UniFrac distances, and Bray–Curtis dissimilarities, are shown in Figure 3. No obvious clustering of the samples among D0, D14, and D28 was observed for any metric. PERMANOVA showed no significant differences in any of the following metrics: weighted UniFrac distances (*p* = 0.471), unweighted UniFrac distances (*p* = 0.157), or Bray–Curtis dissimilarities (*p* = 0.089). In addition, PERMDISP analysis revealed no significant differences in multivariate dispersion among groups for weighted UniFrac distances (*p* = 0.991), unweighted UniFrac distances (*p* = 0.363), or Bray–Curtis dissimilarities (*p* = 0.317).

**Figure 3:**
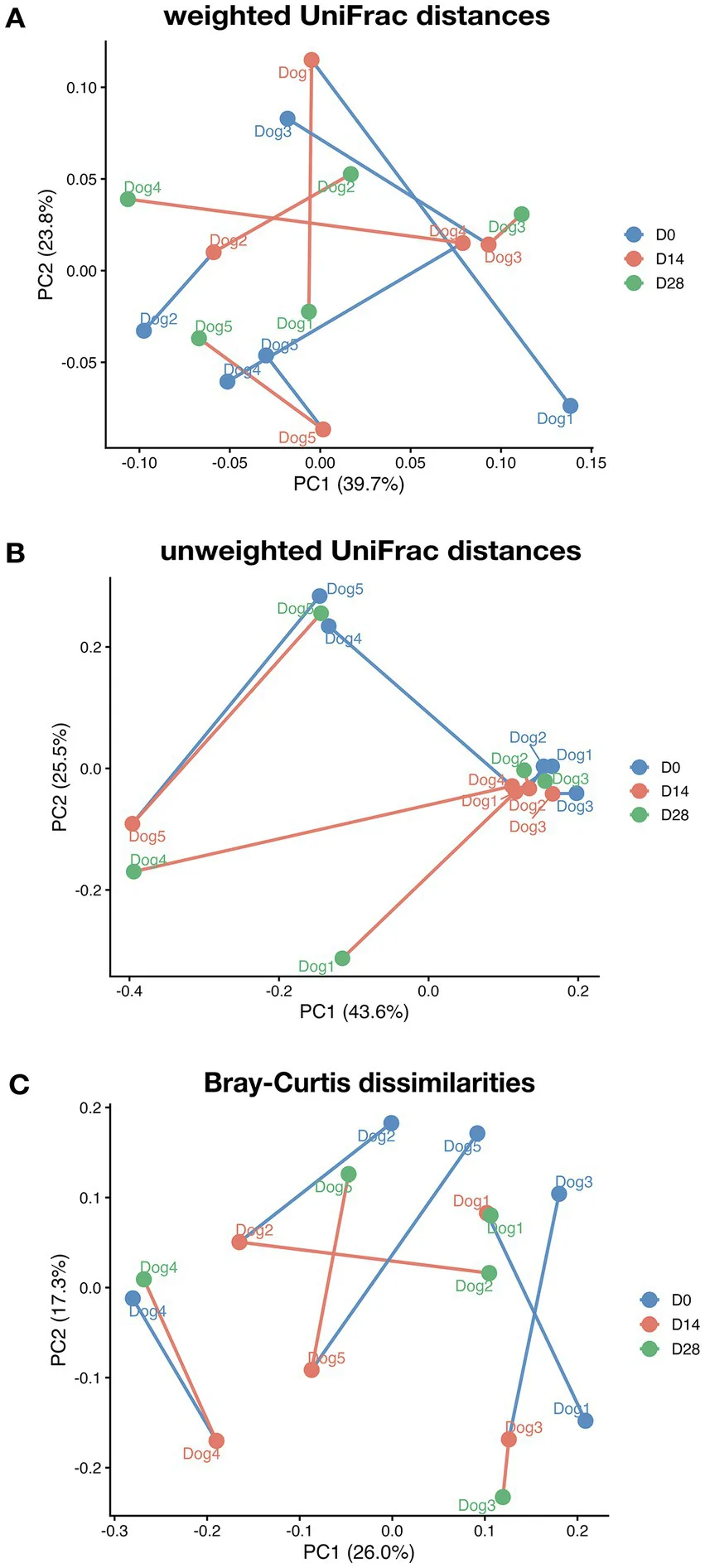
PCoA plots of beta diversity based on **(A)** weighted UniFrac distances, **(B)** unweighted UniFrac distances, and **(C)** Bray-Curtis dissimilarities on day 0 (D0), day 14 (D14), and day 28 (D28). Lines connect samples from the same dog across D0, D14, and D28. Between-group differences were compared using PERMANOVA. No significant clustering of samples among D0, D14, and D28 was observed for any of these metrics.

## 4 Discussion

The aim of our study was to describe fecal microbiota profiles before and during 28 days of rifaximin administration in healthy dogs, as preliminary information for future controlled studies in canine clinical populations. In this small pilot study, no statistically detectable changes were observed in phylum-level composition or alpha and beta diversity metrics during the study period. To our knowledge, this study is the first to evaluate the influence of rifaximin on fecal microbiota in dogs.

Many antibiotics directly affect normal gut bacteria in humans and dogs, leading to an imbalance in the gut microbiota (24–26, 29, 30). This condition is known as antibiotic-induced dysbiosis. In veterinary medicine, dysbiosis has been reported to be associated with acute diarrhea, chronic enteropathy, and obesity (31–33). One study revealed that 14-day administration of metronidazole induced reversible dysbiosis in the gut microbiota of healthy dogs with reduced numbers of butyrate-producing bacteria and bile acid-metabolizing bacteria, such as *Clostridium hiranonis* (25). In addition, metronidazole, alone or in combination with enrofloxacin, has been shown to cause a decrease in the diversity, an increase in the dysbiosis index, and long-term fluctuations in bacterial abundance after discontinuation in dogs (26). Tylosin and amoxicillin-clavulanic acid, commonly used to treat gastrointestinal disorders in dogs, have been reported to reduce bacterial diversity and species richness, increase the dysbiosis index, and have limited efficacy in restoring the gut microbiota (24, 29). Unlike previous studies of some other antibiotics in dogs, the present study did not detect statistically significant changes in fecal microbiota diversity during rifaximin administration. However, because this study did not include a comparator antibiotic or placebo group, direct comparisons with other antibiotics cannot be made.

Rifaximin has a broad spectrum of antibacterial activity against both Gram-negative and Gram-positive bacteria in the gut. In humans, it is used to treat HE, gastrointestinal diseases including traveler’s diarrhea, irritable bowel syndrome (IBS), and SIBO and has been reported to preserve gut microbiota diversity while exerting clinical efficacy (12–15, 17). Previous human studies have investigated the effects of rifaximin on gut microbiota diversity. Rifaximin exerts its antibacterial effects by inhibiting the DNA-dependent RNA polymerase of bacteria, thereby suppressing bacterial RNA synthesis (9, 10). A study administering rifaximin for 8 weeks to patients found no changes in the abundance of the gut microbiota, whereas correlation network analysis revealed reduced connectivity between individual bacteria and metabolites (16). A study administering rifaximin for 12 weeks to patients with HE and cirrhosis suggested that rifaximin maintained the overall diversity of the gut microbiota, but induced specific strain substitutions and genomic mutations, altering metabolic pathways in certain bacteria (18). In our study, no significant changes in gut microbiota diversity were detected after rifaximin administration. These findings appeared to be consistent with previous human studies reporting preservation of the overall gut microbiota diversity during rifaximin treatment. However, our study was designed to evaluate overall changes in the fecal microbiota and did not assess taxonomic changes at lower taxonomic levels or functional alterations of the microbiota; therefore, whether rifaximin affected the functional aspects of the microbiota remains unclear.

Another factor is selectivity for the target bacteria. In the present study, Firmicutes was the dominant phylum before rifaximin administration, while Fusobacteriota, Bacteroidota, and Actinobacteriota were also major phyla. Furthermore, it remained stable for 28 days after rifaximin administration. These compositions are consistent with those described in previous studies that investigated the gut microbiota of healthy dogs (25, 34, 35). In a study of human patients with HE, the target bacteria of rifaximin were species-level pathogenic taxa, such as *Ruminococcus gnavus* and *Streptococcus salivarius* (36). Another report on patients with IBS demonstrated that rifaximin improved clinical signs without altering the gut microbiota composition at the phylum level but by modulating a limited number of low-abundance bacterial species (37). In contrast, rifaximin promotes the growth of beneficial bacteria such as *Bifidobacterium* (38). Because our analysis was limited to phylum-level composition, we could not draw conclusions about changes at lower taxonomic levels or in low-abundance bacterial populations. Future studies using metagenomics and metabolomics are warranted to evaluate changes at lower taxonomic levels and functional alterations of the gut microbiota following rifaximin administration.

In this study, a 28-day course of rifaximin was well tolerated by all dogs. However, this pilot study was not designed to establish the safety, optimal dose, or clinical efficacy of rifaximin in dogs. The rifaximin dosage was based on previous studies evaluating its clinical efficacy in dogs with chronic enteropathy (27). As changes in dosage may have different effects on the gut microbiota of healthy dogs, further investigation is warranted.

Our study had some limitations. First, the small sample size and lack of an untreated control group limited the statistical power of this study and increased the possibility of type II error. The present study was designed as an exploratory within-subject pilot study to describe fecal microbiota profiles before and during rifaximin administration under controlled diet and housing conditions, rather than to compare rifaximin with placebo or other antibiotics. Therefore, placebo and comparator antibiotic groups, such as metronidazole-treated dogs, were not included. The absence of these groups limits causal inference and precludes direct comparison between rifaximin and other antibiotics. Accordingly, the absence of statistically significant differences should not be interpreted as evidence that rifaximin has no effect on the fecal microbiota. Furthermore, all laboratory dogs used in this study were uniform in terms of diet, housing facilities, age, and breed. As these factors can influence the gut microbiota, standardizing them may introduce bias in the microbiota characteristics (39–42). Larger placebo-controlled studies are required to evaluate the effects of rifaximin on the canine fecal microbiota, account for interindividual variability, and determine whether similar results can be obtained under different conditions. Secondly, the experimental period was relatively short. CPSS are the most common cause of HE in dogs, and a minimum stabilization period of 14 days before surgical intervention is recommended for medical management (43). In contrast, chronic liver diseases such as cirrhosis require long-term management, ranging from several months to years, and the treatment duration may have different effects on the gut microbiota. Third, the assessment of antimicrobial resistance gene expression was beyond the scope of our study. Whether the lack of changes in the gut microbiota is influenced by the acquisition of antimicrobial resistance genes by bacteria remains unclear. However, the emergence of rifaximin resistance genes is rare in humans (18). Fourth, fecal samples were stored at −20 °C rather than −80 °C. Although all samples were handled under the same conditions, this storage condition may have affected the recovery of some bacterial taxa.

In conclusion, in this exploratory pilot study of five healthy laboratory dogs, no statistically significant changes in fecal microbiota alpha or beta diversity were detected during 28 days of rifaximin administration. Because of the small sample size, lack of an untreated control group, and limited taxonomic and functional resolution of 16S rRNA gene sequencing, these findings should be interpreted as preliminary. Accordingly, the present microbiome data do not provide evidence to guide clinical use of rifaximin in dogs. Future large-scale placebo-controlled studies incorporating metagenomics and metabolomics are warranted.

## Data Availability

The sequencing data generated in this study have been deposited in the DDBJ database under BioProject accession number PRJDB42512.
